# An Optically Transparent Metamaterial Absorber with Tunable Absorption Bandwidth and Low Infrared Emissivity

**DOI:** 10.3390/ma16237357

**Published:** 2023-11-26

**Authors:** Qi Chang, Jinzu Ji, Wenxing Wu, Yunpeng Ma

**Affiliations:** School of Aeronautic Science and Engineering, Beihang University, Beijing 100191, China; by2105303@buaa.edu.cn (Q.C.); jijinzu@buaa.edu.cn (J.J.); wu_wenxing@buaa.edu.cn (W.W.)

**Keywords:** metamaterial absorber (MMA), tunable, low infrared emissivity, optically transparent

## Abstract

A transparent metamaterial absorber (MMA) with both tunable absorption bandwidth and low infrared (IR) emissivity is proposed in this paper. The MMA is hierarchical, which consists of an infrared shielding layer (IRSL), two radar-absorption layers (RALs), an air/water layer, and an indium–tin–oxide (ITO) backplane from the top downwards. The IRSL and the RALs are made of ITO patterns etched on polyethylene terephthalate (PET) substrates. By changing the thickness of the water, the 90% absorption bandwidth can be tuned from 6.4–11.3 GHz to 12.7–20.6 GHz, while retaining good polarization and angular stability. An equivalent circuit model (ECM) is present, to reveal the physical mechanism of absorption. The proposed MMA has a low theoretical IR emissivity of about 0.24. A sample was fabricated and measured, and the experimental results are consistent with the simulation results, showing its potential applications in stealth glass and multifunctional radome.

## 1. Introduction

With the development of various detection technologies, materials or structures with multispectral stealth capabilities have drawn extensive attention. As radar and infrared (IR) are the two main military detection measures, covering targets with radar–IR bi-stealth materials can greatly improve their survivability in complex situations [1,2,3,4,5]. Many composites with both thermal insulation and microwave absorption effects are proposed for radar–IR bi-stealth, such as Ni-MXene/MF [6], $\mathrm{Fe}/Fe_{2}O_{3}/3D$ carbon [7], and Fe/C/carbon foam [8]. Most of these composites are foam-shaped, and their performance depends on the intrinsic properties of the materials, making it difficult to investigate their internal mechanisms. In addition, the absorption bandwidth of these composites is usually narrow, and the fabrication process is relatively complicated. A more commonly used approach to realizing radar–IR bi-stealth is to cover radar-absorbing materials with low-IR-reflectivity material, such as metal thin films and nanocomposites [9,10,11,12]. However, while radar stealth materials require high absorption and low reflectivity, IR stealth materials require low absorption and high reflectivity, which makes compatible designs very difficult. Moreover, the coverings may damage the original properties of the radar-absorbing materials, resulting in a decrease in absorbing performance [13,14,15].

Metamaterials have powerful electromagnetic control capability, which provides a new approach to the realization of radar–IR bi-stealth materials. Metamaterials can artificially regulate the amplitude, phase, and polarization mode of electromagnetic waves, which allows for great flexibility. As the IR emissivity of metal is generally low, radar–IR bi-stealth materials can be realized by covering metamaterial absorbers (MMAs) with high-metal-filling frequency-selective surfaces (FSSs). Many radar–IR bi-stealth structures have been designed based on this scheme [16,17,18,19,20]. This design only needs to meet the impedance matching between an FSS and an MMA to achieve good performance. Some studies have also designed high-metal-filling checkerboard-shaped metasurfaces, using the principle of electromagnetic wave phase cancellation to reduce electromagnetic scattering in the incident direction, to achieve the radar–IR bi-stealth [21,22,23]. For some applications, such as aircraft windows, that require optical transparency, radar–IR bi-stealth metamaterials using indium–tin–oxide (ITO) and transparent dielectric have been proposed [24,25]. Yang et al. designed a metamaterial window using quartz and ITO, which has a 90% absorption rate at 5.1–19.2 GHz and an IR emissivity of 0.15, while it can retain thermal stability up to 200 ${}^{{}^{\circ}}$C [26]. Zhang et al. proposed a transparent and flexible structure using ITO and polyvinyl chloride, which can achieve 90% absorption at 7.7–18 GHz and maintain stability at 40 ${}^{{}^{\circ}}$C oblique incidence, while the IR emissivity is only 0.23 [27].

IR detectors usually operate in a relatively fixed atmosphere window of 8–14 μm. But with the application of tunable antenna technology, the operating band of the radar may be tunable. In addition, a complex and variable electromagnetic environment also requires materials to have more flexible performance. In response to this situation, many broadband tunable MMAs based on active elements, structural deformation, and tailoring dielectrics have been designed [28,29,30,31,32]. Most reported MMAs have drawbacks, such as a narrow absorption band, low tunability, and being too thick, making it difficult to meet practical needs. Recently, water-based MMAs have attracted much attention, due to their excellent biocompatibility, deformability, and optical transparency [33,34,35]. Water can form a single-medium absorbing structure, or combine with other absorbing materials to form multi-layer absorbing structures. In addition, water-based MMAs can easily achieve tunable absorption by changing the shape, ion concentration, and temperature of water [36,37,38]. Compared to other tunable absorbers, water-based tunable absorbers exhibit superior angle and polarization stability [39,40]. At the same time, water has a large specific-heat capacity, so water-based absorbers theoretically have higher thermal stability. These characteristics mean that water has great application prospects in multifunctional metamaterials.

Most of the previously reported radar–IR bi-stealth MMAs have a fixed structure and operating frequency band, while few studies involve the IR characteristics of tunable MMAs. In this work, we designed a hierarchical MMA structure with both tunable radar absorption bands and low IR emissivity. The structure is made of polyethylene terephthalate (PET), ITO, and water, so it exhibits optical transparency overall. ITO is transparent resistive film with extremely low IR emissivity and microwave absorptivity. Water is a dispersive medium when the incident frequency is lower than 100 GHz. Its dispersion characteristics can be described by the Debye model, and the effective microwave absorption can also be realized after reasonable design [39]. By changing the thickness of the water, tunable broad radar absorption can be achieved. Compared to the previous work published in [41], the proposed MMA exhibited higher tunable bandwidth and angular stability. The surface current distribution of the MMA under different states was studied, to investigate the impact of water on absorption. An equivalent circuit model (ECM) was provided, to reveal the physical mechanism of absorption. Additionally, the angular and polarization stability of the structure was analyzed. Finally, a sample was fabricated, and the radar–IR bi-stealth performance was verified through absorbing-performance experiments and IR imaging.

## 2. Design and Analysis

**Figure 1:**
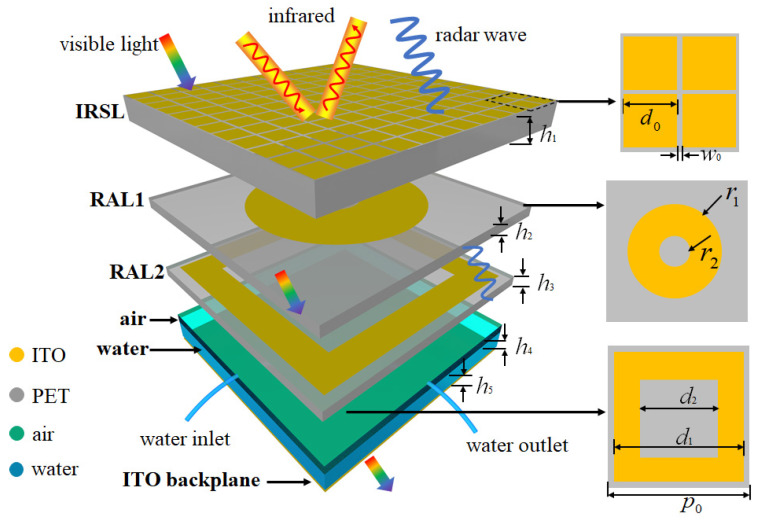
Unit cell of the proposed low-IR-emissivity, tunable, optically transparent MMA structure.

Figure 1 shows a unit cell of the proposed hierarchical structure, which consists of an infrared shielding layer (IRSL), two radar-absorption layers (RALs), air, water, and an ITO backplane from the top to the bottom. The period size of the unit cell is $p_{0}$ = 12 mm. The IRSL was designed with high microwave transmissivity and low IR emissivity. Here, ITO square patches etched on a PET substrate with a thickness of $h_{1}=1$ mm were chosen for the IRSL. The relative permittivity of PET is 3.0(1 − j0.06). The IR radiation intensity of the materials follows the formula $M=e\sigma T^{4}$, where *e* is the IR emissivity and *T* is the temperature. As the temperature is difficult to monitor, the IR radiation intensity of the MMA can be theoretically reduced by reducing the IR emissivity *e*. ITO has low IR emissivity of $e_{\mathrm{ITO}}=0.09$, while PET has high IR emissivity of $e_{\mathrm{ITO}}=0.9$ [18]. The IR emissivity of the IRSL can be calculated as $e_{\mathrm{IRSL}}=e_{\mathrm{ITO}}f_{\mathrm{ITO}}+e_{\mathrm{PET}}\left(1-f_{\mathrm{ITO}}\right)$, where $f_{\mathrm{ITO}}$ is the filling rate of the ITO. Theoretically, the IRSL has the lowest IR emissivity when $f_{\mathrm{ITO}}=1$, but this will make it difficult for radar waves to transmit. The working wavelength of commonly used detection radars is generally higher than 1 cm. When the patch period of the IRSL is less than 5 mm, it can effectively meet the low-frequency radar wave transmission requirements. Therefore, when the size and spacing of the ITO square patches are smaller, the performance of the IRSL is better. However, as the etching accuracy is 0.1 mm, the final optimized ITO patch size is $d_{0}=0.9$ mm, the gap width is $w_{0}=0.1$ mm, and the sheet resistance is 10 Ω/sq, then it can be calculated that $e_{\mathrm{IRSL}}$ is 0.24.

Multilayer absorbing materials usually have a larger absorption bandwidth than single-layer absorbing materials. To obtain broadband microwave absorption, two RALs (RAL1 and RAL2) are used to generate vertically multiple resonances. The ITO pattern of RAL1 is a circular ring with an outer radius of $r_{1}=4$ mm and an inner radius of $r_{2}=1$ mm, and the sheet resistance is 100 Ω/sq. The ITO pattern of RAL2 is a square ring with an external width of $d_{1}=11$ mm and an internal width of $d_{2}=8$ mm, and the sheet resistance is 100 Ω/sq. The thickness of the PET substrates are $h_{2}=h_{3}=0.5$ mm. This structural design allows the structure to generate both horizontal and vertical resonances, thereby improving the overall absorption rate. Air and water with a total thickness of 2 mm are used to control the absorption band, where the air thickness is $h_{4}$ and the water thickness is $h_{5}$. Two pipes are used to control the inlet and the outlet of the water, so the variation range of $h_{5}$ is 0–2 mm. Water is a dispersive medium, and its relative permittivity can be described by the Debye model, which is [39]

(1) $$\varepsilon (\omega ,T)=\varepsilon_{\infty }(T)+\frac{\varepsilon_{s}(T)-\varepsilon_{\infty }(T)}{1-j\omega \tau (T),}$$

where $\varepsilon_{\infty }(T)$, $\varepsilon_{s}(T)$, and τ(T) represent optical permittivity, static permittivity, and rotational relaxation time, respectively. They are all functions of temperature, and the specific function expression can be found in [34]. Equation (1) can accurately describe the electromagnetic characteristics of water when the incident frequency is below 100 GHz. At a room temperature of 25 ${}^{{}^{\circ}}$C, the calculated results of $\varepsilon_{\infty }(T)$, $\varepsilon_{s}(T)$, and τ(T) are 3.1, 78.4, and 8.27 × 10^−12^, respectively.

The backplane is a PET board with a thickness of 0.125 mm coated with ITO film, and the surface resistance of the ITO is 50 Ω/sq. The reflectance R(ω) and transmittance T(ω) of the proposed MMA is simulated by CST Studio Suite 2022 software through S-parameters ${\left|S_{11}\right|}^{2}$ and ${\left|S_{21}\right|}^{2}$, respectively. “Unit cell” and “add open space” boundaries are set in the xy plane and *z* direction, respectively. Then, the absorbance can be calculated as

(2) $$A\left(\omega \right)=1-R\left(\omega \right)-T\left(\omega \right)=1-{\left|S_{11}\right|}^{2}-{\left|S_{21}\right|}^{2}.$$

**Figure 2:**
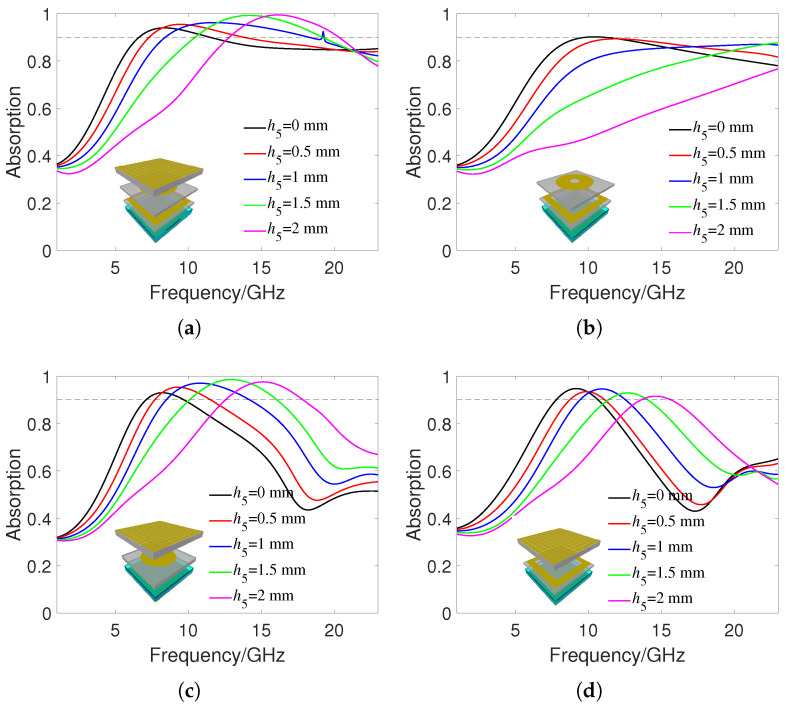
Absorbance under different water thicknesses $h_{5}$: (**a**) The proposed MMA. (**b**) The proposed MMA without IRSL. (**c**) The proposed MMA without RAL1. (**d**) The proposed MMA without RAL2.

Figure 2a shows the absorption rate of the proposed MMA under different water thicknesses $h_{5}$ at a room temperature of 25 ${}^{{}^{\circ}}$C. Due to the appearance of diffraction at higher frequencies, the simulation frequency range here is 0–23 GHz. When $h_{5}$ is 0 mm, the 90% absorption frequency range is 6.4–11.3 GHz. The peak absorption frequency point is 8 GHz, and the relative bandwidth is 55%. As $h_{5}$ increases, both the peak absorption frequency and the 90% absorption bandwidth increase. When $h_{5}$ reaches 2 mm, the 90% absorption frequency range is 12.7–20.6 GHz. The peak absorption frequency and relative absorption bandwidth at this time are 16.2 GHz and 47%, respectively. For comparison, Figure 2b shows the absorbance of the proposed MMA without the IRSL under different $h_{5}$. It can be seen that the peak absorption frequency increases significantly at different water thicknesses. This is because the ITO pattern on the IRSL has better transmission to low-frequency incident waves, thus reducing the resonant frequency of the MMA structure. In addition, the IRSL improves the impedance matching of the overall MMA structure to the free space, ultimately resulting in better wave absorption than MMA without the IRSL. Figure 2c,d show the absorption effect of the proposed MMA with only RAL1 and RAL2, respectively. It can be seen that their absorption effect is smaller than when RAL1 and RAL2 exist at the same time, which verifies the excellent tunable absorbing effect of the proposed MMA. This is because there are significant differences in size and shape between RAL1 and RAL2, resulting in resonance at different frequency points, improving the overall absorption bandwidth.

**Figure 3:**
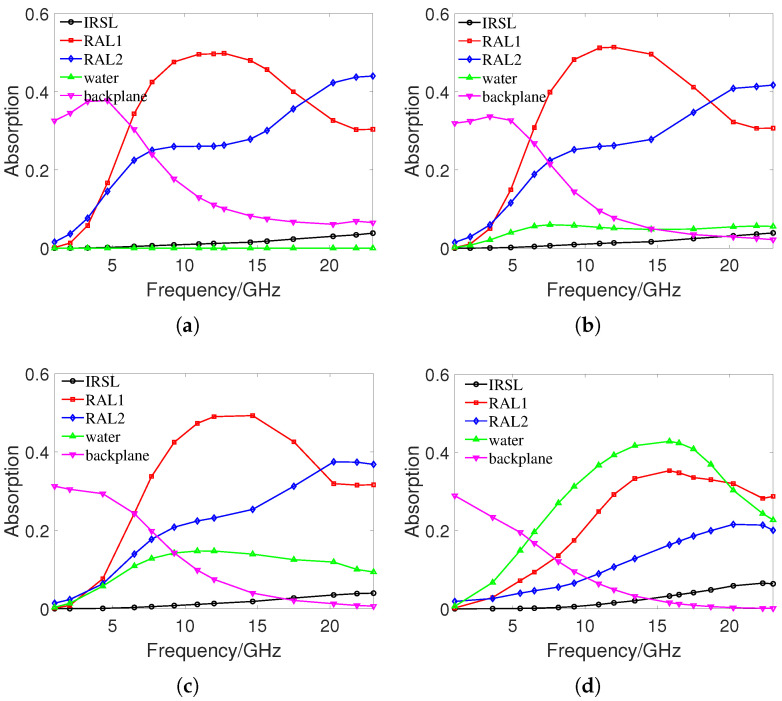
Absorption of each component of the proposed MMA at different $h_{5}$: (**a**) $h_{5}$ = 0 mm. (**b**) $h_{5}$ = 0.5 mm. (**c**) $h_{5}$ = 1 mm. (**d**) $h_{5}$ = 2 mm.

To further investigate the absorption characteristics, Figure 3a–d show the absorption of each component of the proposed MMA at water thicknesses $h_{5}$ of 0, 0.5, 1, and 2 mm, respectively. Because the loss-angle tangent of PET is very small, the absorption effect is negligible. When $h_{5}$ is 0 mm, RAL1 and RAL2 play the leading role in wave absorption, reaching a maximum absorption rate of 50% at 12 GHz and 44% at 23 GHz, respectively. As the frequency increases, the absorption rate of RAL1 first increases and then decreases, and the absorption rate of RAL2 continues to increase. With the increase of $h_{5}$, the absorption rate of RAL1 decreases slightly, but the absorption rate of RAL2 decreases significantly. When $h_{5}$ increases to 2 mm, the absorption of RAL1 and RAL2 increases with frequency. The absorption rate of water increases with the increase of $h_{5}$, and increases first and then decreases with the increase of incident frequency. When $h_{5}$ is 2 mm, the absorption rate of water reaches 42% at 15.9 GHz. With the increase of water thickness, the absorption rate of water is also increasing, and the absorption rate of RAL1 and RAL2 is decreasing. The peak absorption frequency point of RAL1 and water increases with the increase of water thickness. The absorption rate of the IRSL is almost 0 at low incident frequencies, but slowly increases with increasing frequency and water thickness. When the incident frequency is 23 GHz and $h_{5}$ is 2 mm, the maximum absorption rate of IRSL can reach 10%. The absorbance of the ITO backplane decreases with the increase of $h_{5}$ and incident frequency. When the incident frequency is low and $h_{5}$ is 0 mm, the absorption rate of the ITO backplane can reach 37%. When the incident frequency is close to 23 GHz and $h_{5}$ exceeds 1 mm, the wave absorbance of the ITO substrate is almost 0. In general, as the thickness of the water increases, the absorption of water increases, while the absorption of RAL decreases, the maximum absorption frequency point increases, and ultimately the absorption band is shifted to the right.

**Figure 4:**
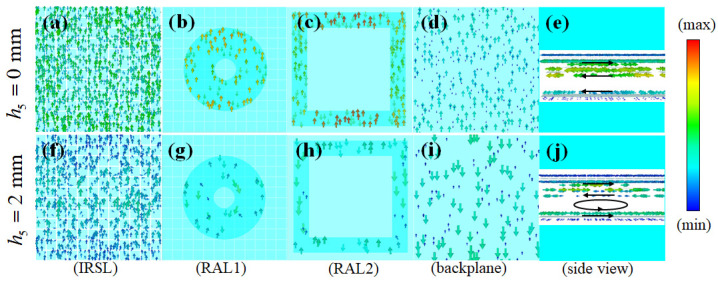
Surface current distribution of MMA at 12.5 GHz when $h_{5}$ is 0 mm and 2 mm: (**a**) IRSL, $h_{5}$ = 0 mm. (**b**) RAL1, $h_{5}$ = 0 mm. (**c**) RAL2, $h_{5}$ = 0 mm. (**d**) Backplane, $h_{5}$ = 0 mm. (**e**) Side view, $h_{5}$ = 0 mm. (**f**) IRSL, $h_{5}$ = 2 mm. (**g**) RAL1, $h_{5}$ = 2 mm. (**h**) RAL2, $h_{5}$ = 2 mm. (**i**) Backplane, $h_{5}$ = 2 mm. (**j**) Side view, $h_{5}$ = 2 mm.

From Figure 2a, we can find that when the incident frequency is 12.5 GHz, the water thickness $h_{5}$ at 0 and 2 mm have almost the same absorption rate. To study the effect of water on the absorption mechanism, Figure 4a–d,f–i show the surface current distribution when $h_{5}$ is 0 and 2 mm, respectively, at the incident frequency of 12.5 GHz. Figure 4e,j show the side views of the surface currents at two water thicknesses. It can be seen that in both cases the surface current on IRSL is opposite to that on RAL1, so a magnetic dipole will be formed, to produce a strong magnetic response. The surface current generated at $h_{5}$ of 0 mm on RAL1 and RAL2 is greater than that generated at $h_{5}$, of 2 mm. The power loss generated at RALs follows the equation of $P_{loss}=I^{2}R$, where *I* is the effective surface current and *R* is the effective surface resistance. Therefore, it can be explained that the absorption rate of RAL1 and RAL2 is larger when $h_{5}$ is 0 mm than when $h_{5}$ is 2 mm. But when $h_{5}$ is 2 mm, the surface currents on RAL2 and the ITO backplane are opposite, thus creating additional magnetic dipoles. This causes the water to absorb a large amount of incident energy, eventually making the absorption rate comparable to that of $h_{5}$ at 0 mm.

**Figure 5:**
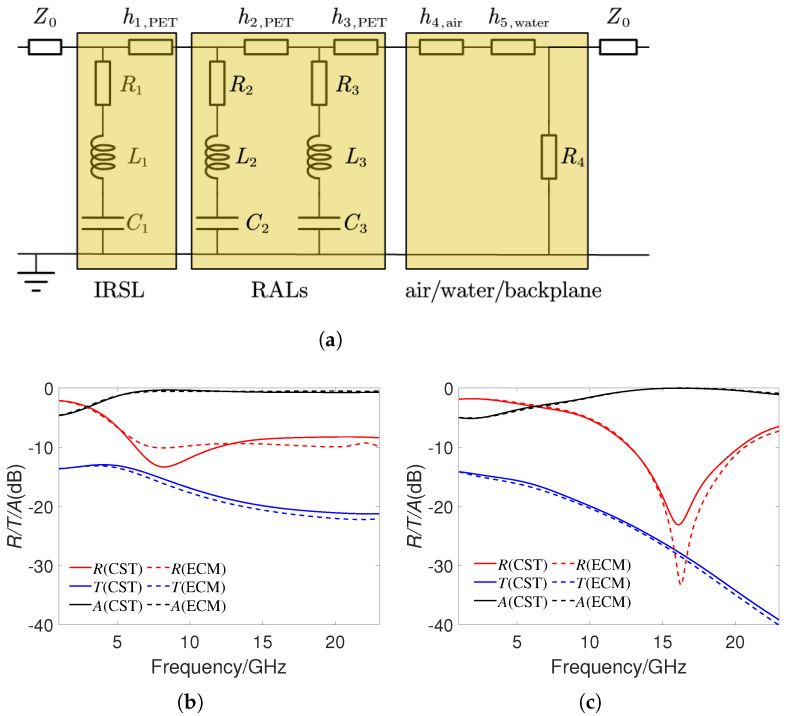
(**a**) The ECM of the proposed MMA structure. (**b**) Comparison of reflection, transmission, and absorption coefficients obtained through CST and ECM when $h_{5}$ = 0 mm. (**c**) Comparison of reflection, transmission, and absorption coefficients obtained through CST and ECM when $h_{5}$ = 2 mm.

An ECM is shown in Figure 5a, to explain the physical mechanism of the MMA structure. The impedance of free space is $Z_{0}=377$ Ω. Considering the situation of normal incidence of electromagnetic waves, the accumulation of charges between adjacent ITO patterned films will generate capacitor, and the effect of surface current will generate inductances. Therefore, ITO patterned films in the IRSL and RALs are equivalent to resistors $R_{n}$, inductors $L_{n}$, and capacitors $C_{n}$, while dielectric layers are equivalent to transmission lines. The impedance of ITO patterned films can be expressed as

(3) $$Z_{n}=R_{n}+j\omega L_{n}+1/j\omega C_{n},n=1,2,3.$$

Then, the ABCD matrix of IRSL, RALs, and air/water/backplane can be represented as (4), where the influence of PET in the backplane is ignored.

(4) $$\begin{array}{c} {\left[\begin{array}{cc} A & B\\ C & D \end{array}\right]}_{\mathrm{IRSL}}=\left[\begin{array}{cc} 1 & 0\\ 1/Z_{1} & 1 \end{array}\right]\left[\begin{array}{cc} \cos(\gamma_{P}h_{1}) & jZ_{P}\sin\left(\gamma_{P}h_{1}\right)\\ j\sin\left(\gamma_{P}h_{1}\right)/Z_{P} & \cos(\gamma_{P}h_{1}) \end{array}\right]\\ {\left[\begin{array}{cc} A & B\\ C & D \end{array}\right]}_{\mathrm{RALs}}=\left[\begin{array}{cc} 1 & 0\\ 1/Z_{2} & 1 \end{array}\right]\left[\begin{array}{cc} \cos(\gamma_{P}h_{2}) & jZ_{P}\sin\left(\gamma_{P}h_{2}\right)\\ j\sin\left(\gamma_{P}h_{2}\right)/Z_{P} & \cos(\gamma_{P}h_{2}) \end{array}\right]\left[\begin{array}{cc} 1 & 0\\ 1/Z_{3} & 1 \end{array}\right]\left[\begin{array}{cc} \cos(\gamma_{P}h_{3}) & jZ_{P}\sin\left(\gamma_{P}h_{3}\right)\\ j\sin\left(\gamma_{P}h_{3}\right)/Z_{P} & \cos(\gamma_{P}h_{3}) \end{array}\right]\\ {\left[\begin{array}{cc} A & B\\ C & D \end{array}\right]}_{a/w/b}=\left[\begin{array}{cc} \cos(\gamma_{a}h_{4}) & jZ_{0}\sin\left(\gamma_{a}h_{4}\right)\\ j\sin\left(\gamma_{a}h_{4}\right)/Z_{0} & \cos(\gamma_{a}h_{4}) \end{array}\right]\left[\begin{array}{cc} \cos(\gamma_{w}h_{5}) & jZ_{w}\sin\left(\gamma_{w}h_{5}\right)\\ j\sin\left(\gamma_{w}h_{5}\right)/Z_{w} & \cos(\gamma_{w}h_{5}) \end{array}\right]\left[\begin{array}{cc} 1 & 0\\ 1/R_{4} & 1 \end{array}\right]. \end{array}$$

In (4), $Z_{P}=Z_{0}/\sqrt{\varepsilon_{\mathrm{PET}}}$ and $Z_{w}=Z_{0}/\sqrt{\varepsilon_{\mathrm{water}}}$ are the characteristic impedance of PET and water, $\gamma_{0}=2\pi /\lambda$, $\gamma_{P}=2\pi \sqrt{\varepsilon_{\mathrm{PET}}}/\lambda$, $\gamma_{w}=2\pi \sqrt{\varepsilon_{\mathrm{water}}}/\lambda$ are the propagating constant of air, PET, and water, where λ represent the wavelength. The total ABCD transfer matrix of the proposed MMA structure is obtained by multiplying the above three matrices. It can be clearly seen that changing $h_{5}$ will change the ABCD matrix of the MMA structure. Using the conversion relationship between the ABCD transfer matrix and S-matrix, we can get the expression of $S_{11}$ and $S_{12}$, then the reflection, transmission, absorption coefficients can be obtained. The RLC parameters calculated through software ADS (https://www.keysight.com/us/en/products/software/pathwave-design-software/pathwave-advanced-design-system.html, accessed on 11 October 2023) are as follows: $R_{1}=10$ Ω, $R_{2}=R_{3}=100$ Ω, $R_{4}=50$ Ω, $L_{1}=0.043$ nH, $L_{2}=1.86$ nH, $L_{3}=1.42$ nH, $C_{1}=3.68$ fF, $C_{2}=0.14$ pF, $C_{3}=0.09$ pF. Figure 5b,c show the reflection, transmission, and absorption coefficients simulated by ECM and CST when $h_{5}$ is 0 and 2 mm, respectively. It can be seen that the calculation results of the two methods are in good agreement, which verifies the effectiveness of ECM.

**Figure 6:**
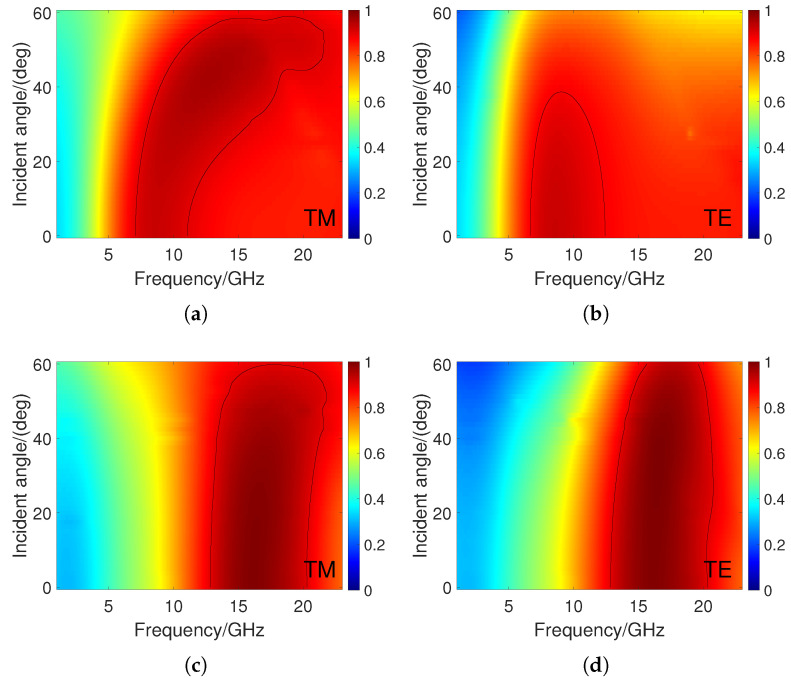
Absorbance under different polarization modes and water thickness: (**a**) TM polarization, $h_{5}$ = 0 mm. (**b**) TE polarization, $h_{5}$ = 0 mm. (**c**) TM polarization, $h_{5}$ = 2 mm. (**d**) TE polarization, $h_{5}$ = 2 mm.

As electromagnetic waves are usually not normally incident, it is important for the MMA to have good polarization and angular stability. Figure 6a,b show the absorption spectrum under TM and TE polarization, respectively, when $h_{5}$ is 0 mm. It can be seen that 90% of the absorption bandwidth can be kept relatively stable in the range of a 0–50${}^{{}^{\circ}}$ incidence angle for TM polarization. However, the absorption frequency range gradually shifts to the right as the incident angle increases. This is because the increase in the incident angle changes the resonance frequencies of RAL1 and RAL2. For TE polarization, the 90% absorption bandwidth narrows as the incident angle increases, and the bandwidth is almost zero when the incident angle exceeds 40${}^{{}^{\circ}}$. This is because the induced current generated by TM polarization is parallel to the MMA, which can more effectively absorb incident waves. For TE polarization, as the incident angle increases, the induced current parallel to MMA continuously decreases, ultimately leading to a decrease in the absorption effect. Figure 6c,d show the absorption spectrum under TM and TE polarization, respectively, when $h_{5}$ is 2 mm. It can be seen that for TM polarization, the absorption effect can maintain stability within the range of 0–60${}^{{}^{\circ}}$ incidence angles at this time. For TE polarization, the angular stability of absorption is also better than that when $h_{5}$ is 0 mm. This may be due to the strong polarity of water molecules, which can generate sufficient induced current even at large incident angles. In addition, as shown in Figure 4, strong magnetic resonance occurs between RALs and ITO substrates in the presence of water, ultimately increasing the polarization stability of the MMA. Compared to the previous work published in [41], the proposed MMA also exhibits better angular stability. From the above analysis, we can see that water can not only improve the absorption bandwidth of MMA, but also increase the angular stability.

## 3. Experimental Verification

A sample consisting of 8 × 8 units was fabricated and measured. The size of the PET boards used for the IRSL and RAL was 110 × 110 mm. ITO films on the PET boards of the IRSL and RALs were first obtained by magnetron sputtering technology, and then an ITO pattern was obtained by laser-etching technology. The actual surface resistance value of ITO film has a processing error of about 5%, but it has little impact on the final result. A 2 mm thick square-ring-shaped PET board, with an outer length of 110 mm and an inner length of 96 mm was used to hold the water, and two small holes were opened laterally, to control the inflow and outflow of water. The layers of the MMA were connected by transparent glue. The thickness of the ITO film was 180 nm, and the total thickness of the MMA was approximately 4.2 mm.

**Figure 7:**
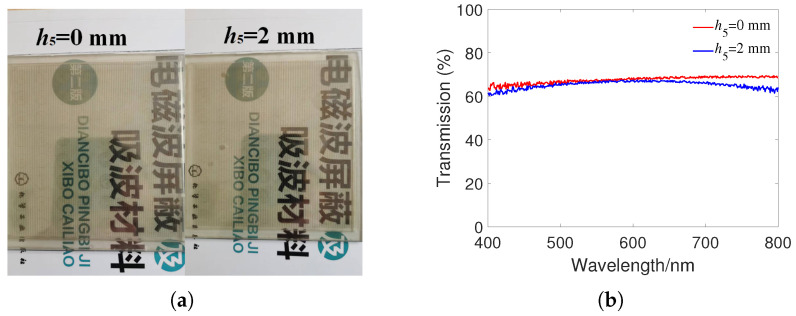
(**a**) Image of the sample when $h_{5}$ is 0 and 2 mm. (**b**) The optical transmittance of the sample in the visible wavelength range of 400–800 nm.

Figure 7a depicts the fabricated sample at $h_{5}$ = 0 mm and $h_{5}$ = 2 mm, and the image below can be clearly seen through the sample, which validates the good transparency of the proposed MMA. Figure 7b shows the optical transmittance of the fabricated sample quantitatively tested by spectrometer (PerkinElmer Lambda 950) in the visible wavelength range of 400–800 nm. It can be seen that the optical transmittance at water thickness $h_{5}$ of 0 and 2 mm was about 65%. Therefore, we can conclude that the fabricated MMA has good optical transparency at different water thicknesses.

**Figure 8:**
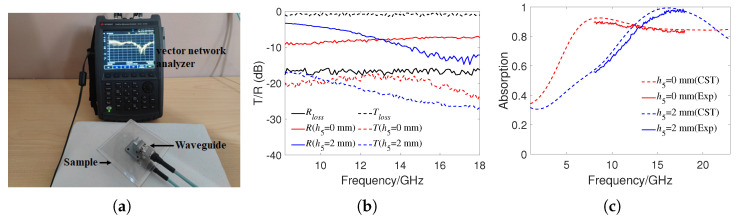
(**a**) Experiment on waveguide method for testing the S-parameters of the sample. (**b**) Experimental reflectivity and transmissivity in three cases of waveguide empty docking, $h_{5}$ = 0 mm and $h_{5}$ = 2 mm. (**c**) Experimental absorptivity results with $h_{5}$ of 0 and 2 mm.

The absorption rate experiment was conducted by the waveguide method. As shown in Figure 8a, two rectangular waveguides were connected to the vector network analyzer (N9918A) through transmission lines, and the sample was sandwiched between two waveguides for S-parameter testing. We used two types of waveguides, WR90 and WR62, and the total operating frequency range was 8.2–18 GHz. Due to the energy loss in the waveguide, the waveguides were first directly docked to test the reflectivity $R_{\mathrm{loss}}$ and transmissivity $T_{\mathrm{loss}}$, then the reflectivity and transmissivity of the waveguide loaded with the MMA were tested. When $h_{5}$ was 0 or 2 mm, the tilt of MMA would not affect the testing and had the highest experimental accuracy. Therefore, we only tested the reflectivity and transmissivity under the conditions of waveguide empty docking, $h_{5}$ = 0 mm and $h_{5}$ = 2 mm. Figure 8b shows the experimental results under three conditions. It can be seen that the transmissivity of the waveguide loaded with the MMA was close to −20 dB, which indicates that most of the incident energy was reflected or absorbed. As the transmittance of the MMA was very low and had relatively large experimental errors, the absorption rate could be directly calculated as A(ω)=1−R(ω). Finally, the true reflectance of the MMA was calculated as $R=R_{\mathrm{MMA}}-R_{\mathrm{loss}}$. The sampling frequency interval of the vector network analyzer was 0.1 GHz. Figure 8c shows the calculated experimental absorptivity and compares it with the simulation results. It can be seen that the experimental results and simulation results have good consistency. Due to the mismatch between the size of the waveguide and the periodic size of the MMA, there existed a certain experimental error. In addition, due to the use of transparent adhesive between the layers of the MMA, there may have been some positional deviation between each layer, and the waveguide and the MMA were not placed absolutely horizontally during testing, all of which may have caused deviations in the experimental results.

**Figure 9:**
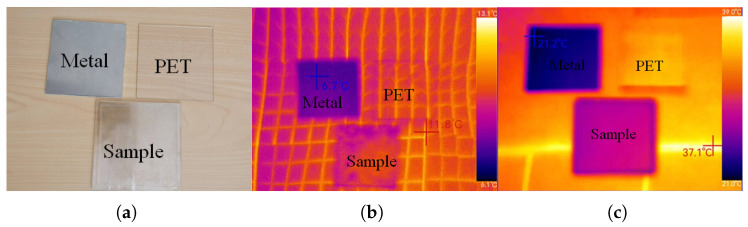
(**a**) Visible light images of the sample, metal plate, and PET plate. (**b**) The IR imaging results at 30 ${}^{{}^{\circ}}$C. (**c**) The IR imaging results at 50 ${}^{{}^{\circ}}$C.

To check the IR property of the proposed MMA, we measured the thermal IR images of the sample by an IR camera (Thermoview T72). The camera works at the IR range of 8–14 μm, and the imaging results of the sample were compared with the imaging results of a metal plate and a PET plate. Figure 9a shows the visible light images of the sample, metal plate, and PET plate. We put the three targets on a heating plate at 50 ${}^{{}^{\circ}}$C for 10 min. Figure 9b,c are the imaging results at 30 ${}^{{}^{\circ}}$C and 50 ${}^{{}^{\circ}}$C, respectively, where higher temperature represents a higher IR emissivity. It can be seen that the IR emissivity of the sample was slightly higher than that of the metal, but significantly lower than that of the PET plate. The differences in the IR emissivity at different positions in Figure 9b,c were caused by inhomogeneous heating. The IR emissivity of the IRSL can be approximately estimated by [42]

(5) $$e_{\mathrm{IRSL}}=\left(T_{r}^{4}-T_{a}^{4}\right)/\left(T_{o}^{4}-T_{a}^{4}\right),$$

where $T_{r}$, $T_{a}$, and $T_{o}$ represent the temperature displayed in the IR image, the ambient temperature, and the actual temperature, respectively. In Figure 9c, $T_{r}$ is approximately 30 ${}^{{}^{\circ}}$C, $T_{a}$ is 25 ${}^{{}^{\circ}}$C, and $T_{o}$ is 50 ${}^{{}^{\circ}}$C. After converting the three temperatures to Kelvin temperature and substituting them into (5), the estimated IR emissivity of the IRSL was 0.18. Due to certain errors in heating temperature, the estimated IR emissivity differed from the theoretical IR emissivity 0.24, but both proved that the IRSL had a very low IR emissivity.

Table 1 shows the performance comparison of the proposed MMA structure with some other reported studies. It can be seen that although the absorption bandwidth of the proposed MMA is relatively narrow, it simultaneously integrates optical transparency, tunable absorption and low IR emissivity. It should be noted that as the height of the water is used to control the absorption band of the MMA, except for the cases of $h_{5}$ = 0 and 2 mm, the absorption effect will be affected when the structure is tilted. In addition, due to the influence of temperature on the relative permittivity of water, the absorption performance of the MMA will also change when the temperature is changed. When the temperature is above 80 ${}^{{}^{\circ}}$C, PET will generate thermal deformation. When the temperature is below 0 ${}^{{}^{\circ}}$C, water will not be in liquid form. Therefore, the proposed MMA havs good performance placed at temperatures within 0–80 ${}^{{}^{\circ}}$C and horizontally.

## 4. Conclusions

In conclusion, an MMA structure with a simultaneously tunable radar absorption band, low IR emissivity and optical transparency was designed, analyzed, and fabricated in this paper. The 90% absorption band of the proposed MMA can be tuned from 6.4–11.3 GHz to 12.7–20.6 GHz by changing the height of water, while maintaining good polarization and angular stability. An ECM was presented to explain the absorption mechanism. Moreover, the MMA showed a theoretical IR emissivity of 0.24. A sample of the proposed MMA was fabricated and measured. The experimental results indicate that the proposed MMA has a transparency of nearly 65% in the visible light spectrum. The absorption experimental results and simulation results were in good agreement, and the IR imaging results verified the lower IR emission. In general, this research demonstrates great potential in applications such as stealth glass and multifunctional radome.

## Figures and Tables

**Table 1:** Comparison of this work to previously reported works.

Reference	90% Absorption Band [GHz]	Relative Bandwidth	Transparency	Low IR Emissivity	Tunability
Ref. [17]	8.1–19.3	82%	No	Yes	No
Ref. [18]	7–12.7	57%	No	Yes	No
Ref. [26]	5.1–19.2	116%	Yes	Yes	No
Ref. [27]	7.7–18	80%	Yes	Yes	No
Ref. [33]	5.8–16.2	94.5%	Yes	No	Yes
Ref. [39]	12.49–98.21	154.9%	Yes	No	Yes
Ref. [41]	7.6–18.2	82.2%	Yes	Yes	Yes
This work	6.4–20.6	105.1%	Yes	Yes	Yes

## Data Availability

The data that support the findings of this study are available from the corresponding author upon reasonable request.
